# Burnout of Greek Teachers: Measurement Invariance and Differences across Individual Characteristics

**DOI:** 10.3390/ejihpe13060079

**Published:** 2023-06-12

**Authors:** Angelos Gkontelos, Julie Vaiopoulou, Dimitrios Stamovlasis

**Affiliations:** 1 School of Philosophy and Education, Aristotle University of Thessaloniki, 54124 Thessaloniki, Greece; 2 Department of Education, University of Nicosia, 2417 Nicosia, Cyprus; 3 School of Psychology, Aristotle University of Thessaloniki, 54124 Thessaloniki, Greece

**Keywords:** OLBI, brnout, psychometric properties, measurement invariance, individual differences

## Abstract

Burnout (BT) is a vital determinant of work effectiveness and a well-studied psychological construct. The dominant theoretical perspectives have defined BT via the proposed dimensional structures and have provided the corresponding instruments for measuring them. The present endeavor adopts the Oldenburg Burnout Inventory (OLBI), as its purpose is to examine the psychometric properties of a short version for the Greek teachers and to find differences across their individual characteristics. The Greek short version of OLBI comprises two dimensions: Disengagement (four items) and Exhaustion (five items), with reliability measures utilizing Cronbach’s alpha and McDonald’s omega: Exhaustion (*α* = 0.810/*ω* = 0.823) and Disengagement (*α* = 0.742/*ω* = 0.756). Confirmatory factor analysis showed an adequate fit of the measurement model: *χ*^2^ = 320.291, *df* = 26, *p* < 0.001; *CFI* = 0.970; *TLI* = 0.958; *RMSEA* = 0.068; 90% CI of RMSEA = [0.062; 0.075]; *SRMR* = 0.067; *NFI* = 0.967; *GFI* = 0.986]. The proposed model is extracted after two studies (*N*_1_ = 134, *N*_2_ = 2437). The novel aspect of this endeavor is the measurement invariance explored across selected demographic variables. The findings supporting the measurement invariance make an essential contribution to the field, and the implications for educational research are provided along with a concise presentation of theoretical issues.

## 1. Introduction

One of the most well-known definitions for burnout (BT) describes it as an exhaustion syndrome, with a simultaneous cynical and negative attitude towards work, as well as reduced professional efficiency, which can appear in any professional activity [1]. In particular, the emotional exhaustion described in the above definition is the central dimension of BT and is expressed through sentimental reactions caused by work. It is followed by a person’s detachment from activities and/or colleagues, while a diminished personal accomplishment is observed that refers to an abatement in one’s feelings regarding his/her ability to complete the task [2]. Many studies have fostered this framework and used the Maslach burnout inventory (MBI) [3] to assess BT, a three-dimensional instrument measuring emotional exhaustion, depersonalization, and (reduced) personal accomplishment. Concomitantly, another instrument—the Oldenburg burnout inventory (OLBI) [4,5]—has been proposed with a different theoretical premise, originating from the jobdemands–resources theory [6,7], which is fostered and examined in the following sections.

### 1.1. Job Demands-Resources Theory

Concurring with the job demands–resources theory [6,7], job characteristics can be classified into two categories: those connected to job demands and those related to job resources. Job demands and available resources have distinctive, independent effects on employees’ well-being and have predictive value [8]. Job demands are those aspects of work that urge the person to exert effort. These include the workload and the difficulty of the assignments, as well as the conflicts that arise in the workplace. Complexity and work burden are necessities that serve as challenges to enhance performance while potentially ensuing conflicts thwart and restrict work performance. 

On the other hand, job resources assist workers in meeting demands to achieve work goals. They are related to the job’s physical, psychological, social, or organizational aspects that aid in achieving work goals, lessen job pressure and expenses, or promote personal development through fulfilling employment [9]. Social support and feedback are two examples of job resources that appear to inspire employees and meet their psychological and emotional requirements while carrying out their professional obligations.

Regarding burnout, situational and personal factors are the two kinds of variables typically used to classify causes of BT [10]. Job demands and (lack of) job resources are situational issues. Job demands are those elements that call for consistent effort [6]. When job demands are high, job resources and skill diversity are the most accurate predictors of work engagement, along with enhanced motivation [8]. Nonetheless, high job demands might have adverse psychological effects because work expectations cause people to feel weary and emotionally distant from their jobs [11].

According to JD–R theory, employees may influence their own job design in one of two ways, depending on their occupational health and well-being: either they are stressed out and negatively affect their working environment, creating a vicious cycle of demands and strain on the job, or they are engaged in their work and positively affect it, by essential engagement and effective use of job resources. Negative job tension results in self-undermining behaviors, which raise job demands and increase job strain. Self-undermined employees report higher levels of workload and emotional pressures, feel more worn out, and receive worse judgment on job performance evaluations from their supervisors [12].

Lower levels of job resources are linked to higher degrees of BT, and this association continuously leads to unfavorable situations, especially when reduced job resources are combined with high job demands [6,10]. This interplay between job resources and job demands might affect the work-related well-being of workers and their performance, even in the short term, by causing them to lose enthusiasm and fulfillment or by enhancing motivation. Research has shown that such temporary fluctuations shape the employees’ relations within the work environment and their productivity. Short-term variations in healthy aspects, such as work engagement, flow, and positive affect, drive a dynamical psychological mechanism that can explain intra-personal changes in behavior, including how people evaluate the undertakings at work and succeed in steadiness between skills and challenges [13]. Employees are usually adaptive to their work environment and confront exhaustion problems due to increasing work demands by expertly utilizing work resources [11]; however, this tradeoff is constantly threatened by imminent burnout situations.

Ergo, it becomes evident that measurement aspects of BT in work environments comprise a crucial issue for valid research and theory development. Within the above-described theoretical framework, the psychometric properties of the Oldenburg burnout inventory (OLBI) [4,5] have been examined and validated for teachers.

### 1.2. The Oldenburg Burnout Inventory

The OLBI encompasses not just the affective characteristics of *exhaustion* but also its physical and cognitive aspects, in contrast to how exhaustion was operationalized in the original MBI. Thus, it is easier to use the tool for both types of workers, those who do physical work and those whose primary responsibility is information processing [14]. The aforementioned notion of cynicism primarily refers to (lack of) interest in the job and occupational worth, whereas depersonalization in the original MBI refers to emotionally separating oneself from service recipients. In a similar line, the OLBI definition of *disengagement* describes a person’s distance from his/her work in general, the work object, and the work content. Additionally, the disengagement items focus on how employees feel about their occupations, particularly their level of identification with their work and inclination to stay in the same line of work. Negative attitudes regarding their job-related goals, work-related material, or work, in general, are shown by disengaged employees. The third MBI dimension, personal accomplishment, is not included in the OLBI as a discrete BT dimension because it is not regarded as an aspect of BT, but it is viewed as a potential side effect of BT and is thought to reflect a personality trait akin to self-efficacy. The OLBI differs from the MBI in that it contains items with both negative and positive wording [14].

Previous studies (Table 1) involving participants from Germany [6,15], Greece [4], and the United States [16] demonstrated good fit indices, supporting the factorial validity of the OLBI as the proposed two-factor model.

### 1.3. Burnout in the School Framework

Regarding schools, due to decreased job satisfaction and an uptick in absenteeism, job BT has a detrimental impact on employees’ occupational health [17,18]. In addition, reduced performance in the classroom has unfavorable effects on learning goals and outcomes [19,20], and further repercussions for education standards. As a result, BT is associated with a weaker classroom climate [17], student disruptive behavior [21], decreased motivation [22], and emotional intelligence [23,24].

The social and emotional standards that govern the teaching profession, teacher–student interactions, parent/colleague disputes, job control, and classroom atmosphere are all stressors associated with BT [25,26]. Compared to the highly demanding didactic task, the limited resources, inefficient school leadership, troublesome student conduct, increased workload, lack of time, and low wages can all contribute to exhaustion and eventual disengagement [27].

Research has co-examined teachers’ BT with their self-efficacy beliefs, where a negative correlation has been identified between the two variables [28,29,30]. Similarly, negative correlations have also been reported between BT and job satisfaction [31,32], teachers’ health [33], psychological empowerment [34], well-being [35], stress [36], and depression [37]. In addition, BT can create individual tendencies to leave and resign from the profession [38], and it can be the final stage of unsuccessfully coping with long-term work-related stress [39]. The utilization of fallout from empirical inquiries is the primary concern of school principals, education stakeholders, and leaders, who should be aware of the origin and processes of burnout.

BT research is currently focused on this issue’s sociodemographic and personal correlates, which may interact with organizational risk factors to either increase or mitigate their impacts [40,41]. As a result of the research on individual differences in this disorder, various investigations of the association of BT with age [1,32] and gender have been conducted [42,43]. Furthermore, researchers are interested in psychological resources for BT healing, such as punctual evaluation, coping, or personality characteristics [41].

## 2. Materials and Methods

### 2.1. Aim of the Study and Research Questions

The study’s main objective is to present a valid version of OLBI and explore potential differences between groups, given the measurement invariance. Thus, the following hypotheses were stated:

- The proposed brief version of OLBI holds satisfactory psychometric properties and factorial validity.
- The two dimensions of burnout, i.e., exhaustion and disengagement, demonstrated satisfactory reliability coefficients.
- There is measurement invariance of the OLBI scale across individual characteristics.
- The two dimensions of burnout, i.e., exhaustion and disengagement, differ across a number of individual characteristics, such as gender, age, years of service, school level, university, and region.

### 2.2. Instrument

The crucial role of BT justifies the importance placed upon implementing advanced statistical modeling to thoroughly examine the multiplicity of the relationships that determine teachers’ behavior, and for that purpose, the present project is dedicated to exploring the psychometric properties of the Greek version of the Oldenburg Burnout Inventory for teachers. OLBI has been translated and used in research with Greek participants [4] but with a different population. The analysis resulted in a shortened version of OLBI, while measurement invariance was examined.

### 2.3. Participants

The data came from teachers who anonymously completed a self-reported questionnaire via an opportunity sampling procedure. It was submitted using a web-based form through Limes-Survey, and an accompanying cover letter highlighted the voluntary involvement, the prospective ambitions, the purpose, and the confidentiality of the study. Emails with the research details and the link to the survey form were sent to the work email addresses of teachers in Greece. Participants replied voluntarily. The data collection technique adhered to the rules established by the Ethics and Deontology Committee of the Aristotle University of Thessaloniki.

A pilot study (*N*_1_ = 134) preceded with teachers, where an exploratory factor analysis was applied to ensure the dimensionality of the instrument and the appropriate ‘items’ loadings. Next, in the main research study, a sample (*N*_2_ = 2437) from the same population was collected, which included 75.6% women, and ages varied from 22 to 68 years old (median = 45, mean = 44.85, SD = 10.33). The years of experience (median = 16.0, mean = 16.39, SD = 10.20), levels of education (15.5% kindergarten, 39.6% elementary school, 22.4%, gymnasium, and 22.5% lyceum), university degrees (36.2% bachelor’s degree, 6.4% double BSc, 52.2% master’s degree and 5.2% Ph.D.), and region (17.9% country, 17.7% town, and 64.4% city) were the additional demographic variables recorded and used for measurement invariance.

### 2.4. Procedures

Principal axis factoring (PAF) was used to conduct exploratory factor analysis (EFA) with promax oblique rotation, and the number of factors was decided based on the screen plot, the Kaiser criterion (eigenvalue > 1), and the percentage of the total variance explained.

In order to establish the validity of the measurement, a confirmatory factor analysis (CFA) was performed on the dataset. CFA is a robust statistical method for explicitly testing the postulated hypotheses regarding the relations between observed variables and their corresponding latent variables. It is a tool often used in the development process of measurement instruments, as it can assess their construct validity and evaluate factor invariance across groups with numerous applications in psychological research [44,45]. Based on the relevant literature, the main fit indexes used were the chi-square (*χ*^2^), the comparative fit index (CFI), the Tucker–Lewis index (TLI), and the root mean square error of approximation (RMSEA). The reliability of the extracted factors was assessed using Cronbach’s alpha (α) and McDonald’s omega (ω).

Following the CFA, measurement invariance was examined to ensure the validity of the Greek short version of OLBI across population subgroups. If the intercepts are not invariant, a bias effect may occur, indicating essential differences across groups regarding the essence of the construct under investigation. Neglecting measurement invariance in instrument development might have severe implications for the conclusions and the generalization of the findings [46]; thus, in psychometric research, there is a growing interest in assessing it (e.g., [47,48,49,50,51,52]. Measurement invariance is a four-step procedure. The first phase assesses *configural invariance* or the least restrictive model used as the baseline. Then, the study moves on to more constrained models, with each step comparing the current model to the prior one. In summary, in the *metric invariance* factor loads are considered equal between groups, but their intersection points are allowed to vary; in the *scalar invariance* model determines whether the item intercepts are equivalent across groups; in the *strict invariance* model, the stability of group variances is examined. The *χ*^2^ difference test is used to compare the invariance models, coupled with the indicative values of Δ*CFI*: 0.01 and Δ*RMSEA*: 0.015, for rejecting the null hypothesis of invariance [44,53].

### 2.5. Data Analysis

The calculations were conducted using R 4.2.1 via the JASP 0.16.4 software. There were no missing data or outliers that needed special treatment.

## 3. Results

### 3.1. Exploratory Factor Analysis (EFA)

The proposed Greek short version of OLBI includes nine items and two dimensions, Disengagement (four items) and Exhaustion (five items) and was created from the original OLBI of 16 items [4,5] via the preceding exploratory procedure (EFA) using a pilot sample from the same population. Not all the initial sixteen items loaded meaningfully with the anticipated factors, and the exploratory procedure resulted in a smaller set of nine items, including those with loadings greater than merely 0.40 that formed a final interpretable structure. Note that the items below the usual threshold of 0.50 were retained to improve the theoretical interpretation and the fit of the latent structure.

**Figure 1:**
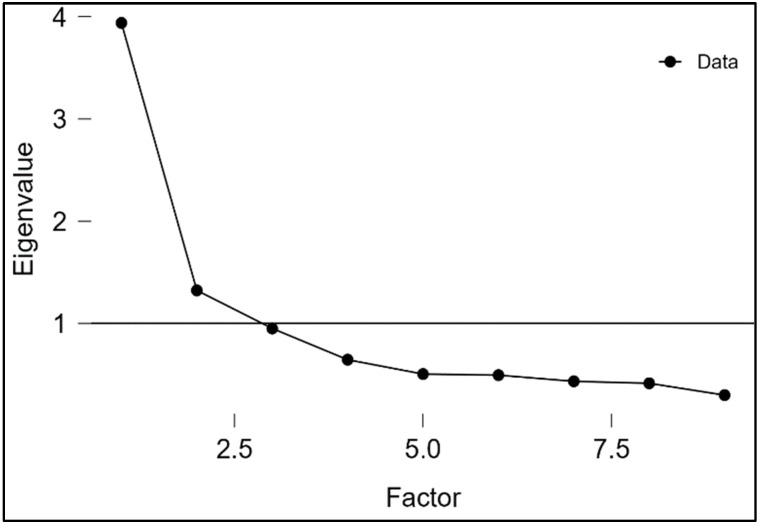
Scree plot showing the two-factor structure.

The Greek short version of OLBI was implemented in the main research, the data of which were used for testing the measurement invariance. The resulting scale comprises two dimensions: *Disengagement* (four items) and *Exhaustion* (five items), and it was used to explore the measurement invariance further. Table 2 and Figure 1 show the factor loading of the items in the two-factor structure and the scree plot. Bartlett’s test of sphericity (*χ*^2^ = 7682.112, *p <* 0.001) and the Kaiser–Meyer–Olkin index (0.851) indicated adequate variance. The eigenvalues for Exhaustion and Disengagement are 3.081 and 1.894, explaining 31.0% and 19.0% of the total variance, respectively.

### 3.2. Reliability Analysis

Reliability measures of the two factors were computed using Cronbach’s alpha (α) and McDonald’s omega (ω): Exhaustion (*α* = 0.810/*ω* = 0.823) and Disengagement (*α* = 0.742/*ω* = 0.756). The overall internal reliability of the Greek short version of OLBI is *α* = 0.838/*ω* = 0.849. The reliability coefficients indicate satisfactory internal consistency (Table 1). The descriptive statistics shown for *Disengagement*: mean = 1.93, SD = 0.689, median = 1.800, mode = 1.40, skewness = 0.940, kurtosis = 0.961, and for *Exhaustion*: mean = 2.660, SD = 0.904, median = 2.75, mode = 2.74, skewness = 0.218, kurtosis = −0.484. The correlation coefficient between the two dimensions is positive, *r* = 0.563, *p* < 0.001. Table 3 shows the items of the short OLBI, means, standard deviations, and the reliability measures of each dimension.

**Table 3:** Items of the Short OLBI.

	Items	Mean	Std. Dev.
**Disengagement** ***a*** = 0.742 ***ω*** = 0.756	D1. I always find new and interesting aspects in my work.	2.04	0.864
	D3(R). It happens more and more often that I talk about my work in a negative way.	1.89	1.077
	D7. I find my work to be a positive challenge.	1.50	0.687
	D9. Over time, one can become disconnected from this type of work.	2.36	1.142
	D11(R). Sometimes I feel sickened by my work tasks.	1.89	1.074
**Exhaustion** ***a*** = 0.810 ***ω*** = 0.823	E4(R). After work, I tend to need more time than in the past in order to relax and feel better.	3.05	1.179
	E8(R). During my work, I often feel emotionally drained.	2.43	1.220
	E10. After working, I have enough energy for my leisure activities.	2.44	0.966
	E12(R). After my work, I usually feel worn out and weary.	2.72	1.153

Note. (R) means reversed item when the scores should be such that higher scores indicate more burnout.

### 3.3. Confirmatory Analysis (CFA)—The Measurement Model

A confirmatory factor analysis (CFA) was carried out with the dataset to assert a valid measurement. CFA results for the single-factor model were: *χ*^2^ = 559.077, *df* = 27, *p* < 0.001, *CFI* = 0.946, *TLI* = 0.928, *RMSEA* = 0.090, *SRMR* = 0.088, *NFI* = 0.943. The two-factor model fitted satisfactorily to the empirical data possessing the following fit measure indices: *χ*^2^ = 320.291, *df* =26, *p* < 0.001; *CFI* = 0.970; *TLI* = 0.958; *RMSEA* = 0.068; 90% CI of *RMSEA* = [0.062; 0.075]; *SRMR* = 0.067; *NNFI* = 0.958; *NFI* = 0.967; *GFI* = 0.986]. A comparison of the two models by means of a *χ*^2^ test revealed that the two-factor model was substantially improved over the single-factor model (Δ*χ*^2^ = 238,787, *df* = 1, *p <* 0.001). Thus, the hypothesis of the unidimensional structure of s-OLBI-G in the present dataset was rejected. Note that the value of TLI is smaller than the value of CFI. TLI is built out of ratios of chi-square over degrees of freedom (*df*), while CFI is built out of differences in chi-square. The model’s *df* affects the magnitude of the difference between the two indexes. However, TLI and CFI are greater than 0.95 and considered satisfactory. Other important indexes are the SRMR (standardized root mean squared residual), which measures the mean absolute correlation residual, and the RMSEA (root mean squared error), which measures the average difference between values predicted by a model and the actual values. Both are measures of ‘non-fit’ [44,45].

**Figure 2:**
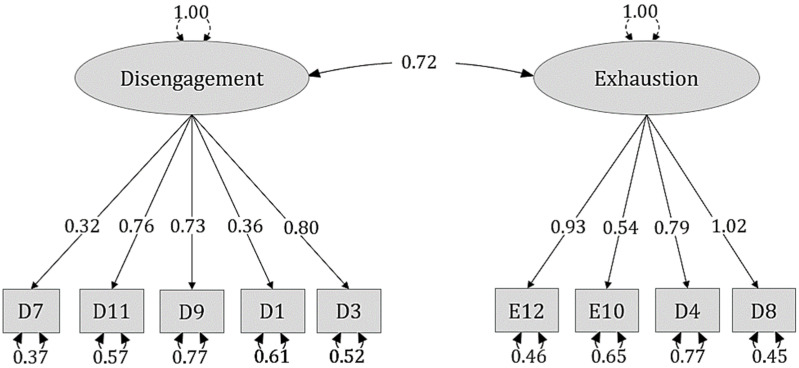
Model plot.

Furthermore, the lack of potential model misspecifications was ensured by inspecting the standardized residual covariance matrix, which had values less than two [45]. Table 4 shows the measurement model, including factors, estimates of factor loadings, standards errors, lower and upper 95% CI and statistical significance. Furthermore, Figure 2 represents the factorial structure of each dimension.

### 3.4. Measurement Invariance for Individual Characteristics

#### 3.4.1. Measurement Invariance for Gender

After completing CFA, measurement invariance was carried out for gender, accordingly to the abovementioned procedure. Table 5 summarizes measurement invariance for the two genders. The chi-square difference (Δ*χ*^2^) test and the differences in the other indexes were used to conclude each invariance model. Starting with evaluating the *configural invariance model*, the next, more restrictive model, the *metric invariance*, has a statistically insignificant *p*-value (*p* = 0.474). This concerns the equality of the factor loadings in the two groups; that is, the meaning of the construct is the same for males and females, and the factor variances and covariances are also certain. The *scalar invariance* shows that the *p*-value was statistically significant. This means that the item intercepts are not equivalent across groups, and thus a bias effect might operate, denoting essential differences across groups in perceiving the essence of BT. For the *strict invariance*, the *p*-value was also statistically significant, i.e., the residual variances are not constant across groups; however, since the overall model fit, based on the other, is not significantly worse compared to the scalar invariance model, it could be considered that constraining the residuals across the two groups does not significantly affect the model fit [54]. The same can also be said for the previous invariance tests for gender. Concussively, although there is a lack of invariance support for some parameters, practically, it does not affect the overall model fit regarding the two genders.

#### 3.4.2. Measurement Invariance for Age and Years of Service

Table 6 and Table 7 show the results of the measurement invariance for age and years of service, respectively. In order to facilitate the analysis, the two interval scale variables (age and years of service) were converted to three-point ordinal variables using a two-step cluster procedure, resulting in three hierarchical categories each. From Table 6, it can be observed that the *metric invariance* has a statistically significant *p*-value, and the same is observed in the *scalar invariance* and *strict invariance* models. This denotes that the equality of the factor loadings, the item intercepts, and residual variances are not equivalent across age groups. This means that the meaning of the construct is not the same across ages, and a bias effect might operate, denoting that older and younger teachers perceive the essence of BT differently. The same can be stated for the variable years of service (Table 7), which is highly correlated with age (*r* = 0.834). However, observing the differences in the other model fit indexes (Δ*CFI* < 0.01; Δ*TLI* < 0.01; Δ*RMSEA* < 0.015; Δ*SRMR* < 0.015), it can be concluded that the differences are negligible and do not affect the total measurement model.

#### 3.4.3. Measurement Invariance for the Region

Table 8 summarizes the results of the measurement invariance for the region. The metric model only has a statistically significant *p*-value (*p* < 0.01), while the rest are insignificant. Taking into consideration the other model fit indexes (ΔCFI < 0.01; ΔTLI < 0.01; ΔRMSEA < 0.015; ΔSRMR < 0.015), the measurement invariance for the region can be supported. Thus, teachers serving in cities, towns, or the countryside possess the same perception of the meaning of the construct in question.

#### 3.4.4. Measurement Invariance for School Level

Table 9 summarizes the results of the measurement invariance for the school level (kindergarten, elementary school, gymnasium, and lyceum). The metric model is statistically insignificant, thus supporting the measurement invariance. Therefore, teachers serving at different levels of education perceive the meaning of the BT construct similarly.

#### 3.4.5. Measurement Invariance for Teachers’ Educational Level

Table 10 summarizes the results of the measurement invariance for teachers’ educational levels. The analysis considered two types of participants, those holding only bachelor’s degrees and those with a post-graduate degree (MSc and/or Ph.D.). The metric model is statistically insignificant, thus supporting the measurement invariance.

### 3.5. Testing the Differences among Individual Characteristics

Given that the measurement invariance among the above individual characteristics was reserved, the underlying differences were tested. Analysis of variance (ANOVA) applied with the school level as an independent variable indicated that teachers in kindergarten had lower Disengagement (*F* = 5.01, *p* < 0.01) compared to teachers in other school levels, while differences in Exhaustion were not statistically significant. Age and years of service were not associated with the two dimensions of BT. Teachers with post-graduate degrees appear to possess a higher degree of Disengagement (*F* = 2.90, *p* < 0.05). Regarding gender, females appear to possess a higher degree of Exhaustion (*F* = 6.24, *p* < 0.01) but a lower degree of Disengagement (*F* = 22.26, *p* < 0.001) compared to males.

## 4. Discussion

Everyday interactions in a work environment and the encompassing emotional problems that influence employees’ views and practices have led to burnout. This effect is accompanied by chronic mental and emotional exhaustion and is associated with the demands involved in their work [55]. In modern society, where the work environment is an integral part of the employee’s daily life, the challenging prevailing working conditions, and occupational stress, combined with economic and social crises, are more likely to affect the emotional state of employees negatively, leading to BT situations that have further consequences for their quality of life.

Education, of course, could not be left unaffected by BT. The teaching profession requires direct contact and cooperation with others, such as students, teachers, parents, and all members and parts of the school organization, and any emotional disturbance can severely affect educational processes [56]. Moreover, educators’ BT has been identified and reported in numerous pieces of research, and teachers are considered the most vulnerable workers, susceptible to burnout [57].

The influential role of BT and its association with important factors of the educational process has sparked research interest in organizational psychology, where the measurement issue becomes crucial. Accessing burnout as a latent variable lies at the center of interest and is a fundamental prerequisite for valid findings and conclusions. This study contributed to this aim by assessing the psychometric characteristics of the Greek short version of the OLBI for teachers (see Appendix A). Exhaustion (E) and Disengagement (D) are the two dimensions in line with the initially proposed structure and the underlying theory, and the CFA model supported the validity of measurement. In addition, an analysis of measurement invariance, which was carried out for a number of individual characteristics, such as gender, age, years of service, school level, university degree, and region, revealed that even though there are differences among the groups under examination, the overall model fit is not threatened. Therefore, the instrument can undoubtedly be implemented for research in the field and definitely can be used to test hypotheses that associate teachers’ burnout with other crucial factors and variables acting in the school environment.

Measurement invariance analysis is a prerequisite to contract research with OLBI, which is a suitable instrument for exploring burnout in different groups and under various conditions. Gender, for example, has been reported as a susceptibility factor for burnout, which influences women more than men [20,42]. In addition, past and more recent studies [1,58] have pointed out disparities between the different age groups, while professional experience in terms of years of service also appears to affect teachers differently [8].

Certain study limitations, of course, originate from the cross-sectional design with a self-reported questionnaire. An additional issue could be the opportunity-sampling procedure, which included participants that were willing to respond; nonetheless, this concern may be overcome by the sufficiently large sample.

Finally, using the Greek short version of the OLBI will endorse new research on the organizational problems and issues of teachers’ behavior. The inhibitory role of burnout in teachers’ productivity gives BT primacy when probing the failures and disappointments in the school framework, along with co-exploration with other variables, such as self-efficacy, irrational beliefs, innovative behavior, and creativity, and therefore the avenues of inquiry are growing. Moreover, valid measurements via the Greek short version of the OLBI will facilitate further investigations and explorations of potential nonlinear phenomena induced by the dynamic interactions between job resources and job demands [59], where BT has a determinant role in the teachers’ work-related well-being and performance.

Moreover, the results from ANOVA indicate some individual differences: the fact that teachers in kindergarten, even though they do not differ in Exhaustion, possess lower Disengagement could be interpreted by the higher emotional engagement that they might have with younger kids. It is somewhat unanticipated that teachers with post-graduate studies possess a higher degree of Disengagement. However, the effect of gender is undoubtedly interpretable in agreement with previous research [60]; that is, females, even though they possess a higher degree of Exhaustion, have lower Disengagement, possibly because of their stronger emotional engagement with children. The above findings and the differences identified confirm that BT’s dimensions are distinct and should be studied separately.

### Practical Implications

The results of the present endeavor reinforce the underpinning framework by adding to both the measuring processes and the theoretical development of BT within organizational psychology, the implications of which concern predominately the school leadership. As was mentioned in the preceding sections, leaders share responsibility for the overall advancement of the organization and managing the welfare of the employees. Leaders should be aware of the theoretical underpinnings that explain workers’ behavior, specifically the JD–R model, which suggests effective ways to face the interplay between job demands and job resources and the behavioral patterns that might lead to burnout experience. Treating and appeasing BT requires prevention. Often, it has been observed that drastic measures are taken after work stress has reached high levels, whereas suitable interventions could keep employees motivated and thus inventive and efficient [61,62]. Besides this, the reduction in job demands and the expansion of job resources, combined with the adaptation of the work object according to the individual’s preferences and abilities, seems to favor the overall development of the school. In addition, informed leadership should also be mindful that the phenomena under investigation might not be linear, and the changes could be abrupt, making potential burnout hard to control [59,63].

## 5. Conclusions

The present endeavor’s main contribution is validating the Greek short version of the OLBI, a scale with satisfactory psychometric properties, to assess teachers’ burnout. Especially with the preserved measurement invariance across individual characteristics, the instrument becomes a powerful tool for ensuring the validity of future investigations at all levels of education. The first analysis, comparing the degree of burnout among educators, showed that the two dimensions operate differently, with Disengagement appearing independently from the degree of Exhaustion. The findings, even though interpretable, suggest further investigations for a better understanding of the phenomenon of burnout and its implications for teachers’ effectiveness.

## Figures and Tables

**Table 1:** Previous research for the OLBI’s factorial validity.

Country	Participants	Reliability	Correlation	Fit Indices
Germany [6]	374	Exhaustion’s a = 0.82 Disengagement’s a = 0.83	*r* = 0.39 ***	*χ*^2^ = 109.55, *p* = 0.004, *df* = 73, *GFI* = 0.96, *RMR* = 0.04, *NFI* = 0.94, *CFI* = 0.98, *IFI* = 0.98
Germany [15]	294	Exhaustion’s *α* = 0.85 Disengagement’s *α* = 0.84	*r* = 0.39 ***	*χ*^2^ = 166.87, *p* = 0.022, *df* = 132, *GFI* = 0.93, *RMR* = 0.05, *NFI* = 0.91, *CFI* = 0.99, *IFI* = 0.98
Greece [4]	232	Exhaustion’s *α* = 0.73 Disengagement’s *α* = 0.83	*r* = 0.44 ***	*χ*^2^ = 217.04, *p* = 0.001, *df* = 64, *GFI* = 0.87, *RMSEA* = 0.10, *NFI* = 0.78, *CFI* = 0.83, *IFI* = 0.84
The United States [17]	2431	Exhaustion’s *α* = 0.74 Disengagement’s *α* = 0.76	*r* = 0.32 ***	*χ*^2^ = 112.7, *p* = 0.001, *df* = 103, *GFI* = 0.97, *RMSEA* = 0.03, *NNFI* = 0.96, *CFI* = 0.95

Note. *** *p* < 0.001.

**Table 2:** EFA—Principal Axis Factoring (PAF), with oblique—promax rotation. (The questions are presented in Table 3).

Factor Loadings			
	Exhaustion	Disengagement	Uniqueness
E12	0.958		0.264
E4	0.925		0.656
E8	0.664		0.568
E10	0.506		0.664
D9		0.623	0.787
D11		0.602	0.614
D3		0.595	0.584
D1		0.493	0.553
D7		0.448	0.313
Eigenvalues	3.081	1.894	

Note. The applied rotation method is promax.

**Table 4:** CFA measurement model: Factors, estimates of factor loadings, standards errors, lower and upper 95% CI, and statistical significance.

						95% Confidence Interval	
Factor	Indicator	Estimate	Std. Error	z-Value	*p*	Lower	Upper
**Disengagement**	D3	0.793	0.021	37.817	<0.001	0.752	0.834
	D11	0.768	0.021	36.462	<0.001	0.727	0.810
	D9	0.699	0.023	30.074	<0.001	0.653	0.744
	D1	0.380	0.018	20.529	<0.001	0.343	0.416
	D7	0.340	0.014	23.476	<0.001	0.312	0.369
**Exhaustion**	E12	0.974	0.020	47.719	<0.001	0.934	1.014
	E8	0.938	0.022	41.918	<0.001	0.894	0.982
	E4	0.836	0.022	37.687	<0.001	0.792	0.879
	E10	0.545	0.019	28.280	<0.001	0.508	0.583

**Table 5:** Measurement Invariance for Gender.

Invariance Model	*χ* ^2^	*df*	CFI	TLI	RMSEA	SRMR	Δ*χ*^2^	Δ*df*	*p*-Value
	0	0							
Configural	337.129	52	0.971	0.960	0.067	0.068	337.13	52	
Metric	343.716	59	0.971	0.965	0.063	0.068	6.587	7	0.473
scalar	365.152	66	0.970	0.967	0.061	0.063	21.436	7	<0.01
Strict	382.18	75	0.969	0.970	0.058	0.066	17.028	9	<0.05

**Table 6:** Measurement Invariance for Age.

Invariance Model	*χ* ^2^	*Df*	CFI	TLI	RMSEA	SRMR	Δ*χ*^2^	Δ*df*	*p*-Value
	0	0							
Configural	350.701	78	0.973	0.962	0.066	0.070	350.7	78	
Metric	384.06	92	0.971	0.965	0.063	0.073	33.359	14	<0.01
scalar	464.393	106	0.964	0.963	0.065	0.071	80.333	14	<0.001
Strict	490.424	124	0.963	0.968	0.060	0.073	26.031	18	0.099

**Table 7:** Measurement Invariance for Years of Service.

Invariance Model	*χ* ^2^	*Df*	CFI	TLI	RMSEA	SRMR	Δ*χ*^2^	Δ*df*	*p*-Value
	0	0							
Configural	339.161	78	0.974	0.963	0.064	0.069	339.16	78	
Metric	367.687	92	0.972	0.967	0.061	0.071	28.526	14	<0.05
scalar	438.721	106	0.966	0.966	0.062	0.069	71.034	14	<0.001
Strict	471.991	124	0.965	0.969	0.059	0.072	33.27	18	<0.05

**Table 8:** Measurement Invariance for Region.

Invariance Model	*χ* ^2^	*Df*	CFI	TLI	RMSEA	SRMR	Δ*χ*^2^	Δ*df*	*p*-Value
	0	0							
Configural	337.141	78	0.974	0.964	0.064	0.068	337.14	78	
Metric	372.398	92	0.971	0.967	0.061	0.071	35.257	14	<0.01
scalar	384.681	106	0.972	0.971	0.057	0.066	12.283	14	0.58
Strict	400.469	124	0.972	0.976	0.052	0.068	15.788	18	0.60

**Table 9:** Measurement Invariance for school level.

Invariance Model	*χ* ^2^	*Df*	CFI	TLI	RMSEA	SRMR	Δ*χ*^2^	Δ*df*	*p*-Value
	0	0							
Configural	349.408	104	0.975	0.965	0.062	0.070	349.41	104	
Metric	376.894	125	0.974	0.971	0.058	0.072	27.486	21	0.15
scalar	407.89	146	0.973	0.974	0.054	0.067	30.996	21	0.073
Strict	436.472	173	0.973	0.978	0.050	0.071	28.582	27	0.38

**Table 10:** Measurement Invariance for teachers’ educational level.

Invariance Model	*χ* ^2^	*Df*	CFI	TLI	RMSEA	SRMR	Δ*χ*^2^	Δ*df*	*p*-Value
	0	0							
Configural	330.389	52	0.972	0.961	0.066	0.068	330.39	52	
Metric	340.427	59	0.971	0.965	0.063	0.069	10.038	7	0.18
scalar	351.163	66	0.971	0.968	0.060	0.064	10.736	7	0.15
Strict	355.658	75	0.971	0.973	0.055	0.064	4.495	9	0.87

## Data Availability

The data presented in this study are available on request from the corresponding author.

## Appendix A

**Table:**

Oldenburg Burnout Inventory				
	Strongly agree	Agree	Disagree	Strongly disagree
**1.** **I always find new and interesting aspects in my work.**	1	2	3	4
2. There are days when I feel tired before I arrive at work.	1	2	3	4
**3.** **It happens more and more often that I talk about my work in a negative way.**	1	2	3	4
**4.** **After work, I tend to need more time than in the past in order to relax and feel better.**	1	2	3	4
5. I can tolerate the pressure of my work very well.	1	2	3	4
6. Lately, I tend to think less at work and do my job almost mechanically.	1	2	3	4
**7.** **I find my work to be a positive challenge.**	1	2	3	4
**8.** **During my work, I often feel emotionally drained.**	1	2	3	4
**9.** **Over time, one can become disconnected from this type of work.**	1	2	3	4
**10.** **After working, I have enough energy for my leisure activities.**	1	2	3	4
**11.** **Sometimes I feel sickened by my work tasks.**	1	2	3	4
**12.** **After my work, I usually feel worn out and weary.**	1	2	3	4
13. This is the only type of work that I can imagine myself doing.	1	2	3	4
14. Usually, I can manage the amount of my work well.	1	2	3	4
15. I feel more and more engaged in my work.	1	2	3	4
16. When I work, I usually feel energized.	1	2	3	4

*Note.* **Bold Items Remained in the final model.** Disengagement items are 1, 3 (R), 6 (R), 7, 9 (R), 11 (R), 13, 15. Exhaustion items are 2 (R), 4 (R), 5, 8 (R), 10, 12 (R), 14, 16. (R) means that reversed items when the scores should be such that higher scores indicate more burnout.
